# The Upregulation of PLXDC2 Correlates with Immune Microenvironment Characteristics and Predicts Prognosis in Gastric Cancer

**DOI:** 10.1155/2021/5669635

**Published:** 2021-11-05

**Authors:** Yang Li, Jia-qi Li, Hai-ping Jiang, Xin Li

**Affiliations:** ^1^Department of Clinical Nutrition, The First Affiliated Hospital of Jinan University, Guangzhou, Guangdong, China; ^2^Department of General Surgery, The First Affiliated Hospital of Jinan University, Guangzhou, Guangdong, China; ^3^Department of Gastrointestinal Surgery, The First Affiliated Hospital of Jinan University, Guangzhou, Guangdong, China; ^4^Clinical Innovation & Research Center, Shenzhen Hospital of Southern Medical University, Shenzhen, Guangdong, China

## Abstract

Tumor microenvironment (TME) has been demonstrated to exhibit a regulatory effect on the progressions of gastric cancer (GC). However, the related functions of stromal and immune components (TME-associated genes) in TME remain largely unclear. From the TCGA dataset, we downloaded the clinical data of 375 GC cases and then estimated the percentage of tumor-infiltrating immunocytes (TICs) and the levels of immune and stromal constituents by the use of CIBERSORT and ESTIMATE tolls. Univariate assays were applied to study the differentially expressed genes. The associations between the clinical information of GC patients and the expressions of the specific genes were analyzed based on the TCGA datasets. The effect of Plexin domain containing 2 (PLXDC2) expression on TICs was conducted. We observed that PLXDC2 expression was distinctly upregulated in GC specimens compared with nontumor gastric specimens. Its upregulation was associated with advanced clinical stages and predicted a shorter overall survival of GC patients. The genes in the group of higher expressing PLXDC2 were primarily enriched in immunity-associated events. By the use of CIBERSORT, we observed that PLXDC2 expressions were related to the proportion of dendritic cells resting, T cell CD4 memory resting, eosinophils, mastocyte resting, mononuclear cells, plasma cells, T cell follicle helper, macrophage M2, and dendritic cells activated. Overall, our discoveries revealed that the expression of PLXDC2 was remarkable in GC, might be a possible biomarker for GC, and provided novel contents regarding immune infiltrates, offering novel insight for treatments of GC.

## 1. Introduction

Gastric cancer (GC) is the 2^nd^ most common cause of tumor-associated morality and the 4^th^ most commonly seen tumor across the globe [1, 2]. The prevalence and mortality of which are also significantly higher in developing countries than that in developed countries [3]. Despite the fact that gastroscope has facilitated the decrease of such diseases via allowing early detection of GC, the majority of sufferers are confirmed at late period and exhibit inferior prognostic results [4, 5]. Conventionally, the TNM stage was employed as a marker to forecast the prognostic results of sufferers, and recently, researchers have evidenced that merely the standards are not adequate enough to estimate prognostic results [6, 7]. Hence, it is pivotal to explore the molecule causal links pertaining to the GC carcinogenetic process and determine diagnosis biomarkers for early diagnosis and target therapy of GC.

The progression of tumors is considered to be a sophisticated process involving various noncell and cell constituents in the tumor microenvironment (TME) [8]. TME serves as an essential component in tumor developments, which contains nonmalignance cells like bone marrow-derived cells, extracellular matrix, endotheliocytes and adventitial cells, tumor-related fibroblasts, and immunocytes and inflammation cells [9, 10]. The potential functions of TME in response to antiangiogenic therapy or chemotherapy have been noted in recent years [11]. The activation, proliferation, and migration of fibroblasts serve as a positive regulator in the wound healing process for healthy specimens [12]. However, tumor-related fibroblasts exhibited a positive effect on tumor growth and metastasis, suggesting its important effects on the modulation of tumor progression. More and more evidence has demonstrated that the prime constituents of TME (infiltrating stroma cells and immunocytes) acted as important players in tumor progression [13, 14]. In recent years, more and more checkpoint-blocking drugs were developed, and several of them have been used for tumor treatments in clinical practice, such as anti-PD-1, anti-PD-L1, and anti-CTLA-4 [15–17]. Thus, identification of novel immune-associated therapeutic targets is necessary for the developments of immune treatment.

In this research, our team performed a variety of bioinformatic analyses and identified several genes involved in the activity of immunocytes and stroma cells. As per the above genes, we identified 26 prognostic genes. Importantly, among them, we focused on Plexin domain containing 2 (PLXDC2) which is a component of the tumor endothelial marker (TEM) family. We provided proof that PLXDC2 might be a novel marker for the prognostic results of GC sufferers and a novel therapeutic target of GC.

## 2. Materials and Methods

### 2.1. Patients and Specimens

Paired samples (cancer and nontumor specimens) from 9 patients with GC were obtained from The First Affiliated Hospital of Jinan University, between January 2019 and December 2020. All were immediately frozen and then stored at -80°C for RT-PCR assays. The histopathological diagnosis of all samples was, respectively, diagnosed by two pathologists. The informed consents were provided by all the patients. All of the experiments were approved by the Ethics Board of The First Affiliated Hospital of Jinan University.

### 2.2. Cell Culture and Transfection

GC cell lines (MKN28, MKN45, BGC823, HGC27, and SGC7901) and the GES-1 were obtained from the type Culture Collection of Chinese Academy of Sciences (Shanghai, China). Cells were cultured in DMEM with 10% Gibco fetal bovine serum (GIBCO, Guangzhou, Guangdong, China) at 37°C in 5% CO_2_ control. To generate PLXDC2 knockdown GC cells, the target sequence for PLXDC2 siRNA (si-PLXDC2-1 and si-PLXDC2-2) or scrambled siRNA (si-NC) that did not correspond to any human sequence was synthesized by Invitrogen. According to the manufacturer's protocol, the transfection was carried out by applying the Lipofectamine 2000 transfection reagent (Invitrogen, China).

### 2.3. Data Collection

The mRNA expressing data and clinic information of GC sufferers were provided by TCGA [18]. We searched the ImmPort data center (http://www.immport.org) which was employed to determine DEGs, providing the ability to screen the genes involved in immunologic processes.

### 2.4. DEG Identification

The edgeR package was applied to screen GC-related DEGs via comparing expressing profiles of genes in 375 cancer and 32 nontumor samples. DEGs were determined using the following standards: FDR < 0.05, ∣log2 (FC) | >1. “GEPIA” was applied to confirm the dysregulated genes between cancer specimens and non-tumor specimens from the Genotype-Tissue Expression (GTEx) and TCGA datasets [19].

### 2.5. Computation of Stroma Value, Immunity Value, and ESTIMATE Value

Our team utilized ESTIMATE arithmetic to compute the percentage of immunity and stromal components in TME for every specimen, which was described as the immunity value and stroma value. The ESTIMATE value denotes the sum of immunity value and stroma value. The 3 types of values were related to the percentage of stroma, immunity, and the sum of the first two in a positive way, separately.

### 2.6. Heatmaps

Thermographs of DEGs were generated by R language with package “pheatmap.”

### 2.7. Function Enrichment Assay

The bioinformation assay approaches were similar to the investigation approaches aforementioned herein. At the same time, our team utilized the STRING tool to perform the function protein correlation networks [20]. To delve into the functional annotation for DEGs, we used GO and Kyoto Encyclopedia of Genes and Genomes (KEGG) enrichment analyses.

### 2.8. Survival and Hazard Analyses

The “survival” package 3.2-7 of R software was employed for univariable Cox regressive assay of the TCGA specimens. Genes which passed the univariable Cox test are presented by the illustration. PLXDC2 expressions were separated into PLXDC2 high and PLXDC2 low groups as per the medium expression score. Kaplan-Meier assays were employed to explore the possible differences between high and low PLXDC2 expressing groups. The clinical characteristics of PLXDC2 were explored when comparing with PLXDC2 expressions by the use of Wilcoxon rank-sum test.

### 2.9. TICs Profile

CIBERSORT methods were conducted to examine the TIC abundance profiles in cancer specimens before quality filtration that 375 cancer specimens with *p* < 0.05 were screened for further assay [21].

### 2.10. RNA Extraction and Quantitative Real-Time PCR

The TRIzol reagent (Invitrogen, China) was applied to extracted total RNA. RNA concentration was examined with a NanoDrop ND-2000 spectrophotometer (Life Technologies). Quantitative real-time PCR (qPCR) was carried out by the application of the SYBR Green quantitative PCR kit (Takara, Hangzhou, Zhejiang, China) using the 7500 Real-Time PCR System. The results were normalized to the expression of GAPDH. The primers were as follows: GAPDH sense 5′- ACAACTTTGGTATCGTGGAAGG-3′, reverse 5′- GCCATCACGCCACAGTTTC-3′ and PLXDC2 sense, 5′- CCAGTTTCAGTTCGCCGATG-3′, reverse 5′- TGTCTACCGCCTTGAGAAAGT-3′. The qRT-PCR data were analyzed and calculated using the 2^−*ΔΔ*Ct^ methods.

### 2.11. CCK-8 Assay

CCK-8 (Dojindo, Pudong, China) was applied for cellular proliferation. In 96-well plates, cells were seeded at a density of 3 × 10^3^ cells. After culture for 0, 24, 48, and 72 h, 15 *μ*l CCK-8 reagent was added to each well, followed by incubation for another two hours. A plate reader was applied to examine optical density values at 450 nm.

### 2.12. Statistical Assay

R software 3.6.3 was applied for all statistical analyses. Wilcoxon rank-sum tests or Kruskal-Wallis tests were employed to perform comparisons between different groups. Kaplan-Meier assays were applied to conduct survival assays. Univariate assays were used to demonstrate the independent factors. *p* scores < 0.05 had significance on statistics.

## 3. Results

### 3.1. Scores Were Correlated with the Survival of GC Patients

**Figure 1:**
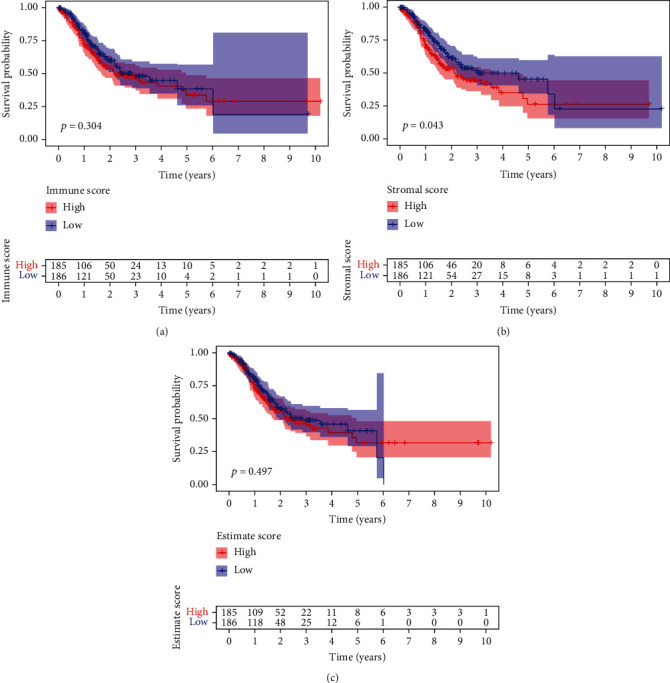
The association of scores with the survivals of GC cases. (a) Kaplan-Meier assays for GC cases based on low or high scores in ImmuneScore. (b) Survival analysis for StromalScore. (c) Survival analysis for GC cases grouped by ESTIMATEScore.

ESTIMATES arithmetic was adopted to assess the percentage of immunity and stroma components for TCGA-STAD sufferers. Our team separated GC sufferers into the high- and low-score group as per the medium score immunity value, stroma value, and ESTIMATE value. In addition, Kaplan-Meier analysis was employed to determine the relationship of the fraction of immunity and stroma constituents with the survival possibility. As presented by Figure 1(a), ImmuneScore exhibited no remarkable relationship with the OS ratio of GC patients. However, the percentage of StromalScore positively exhibited an association with the OS ratio of GC patients (Figure 1(b)). ESTIMATEScore did not show distinct association with overall survival of GC patients (Figure 1(c)).

### 3.2. Association of Immunity Value and Stroma Value with the Clinicopathology Factors of GC Cases

**Figure 2:**
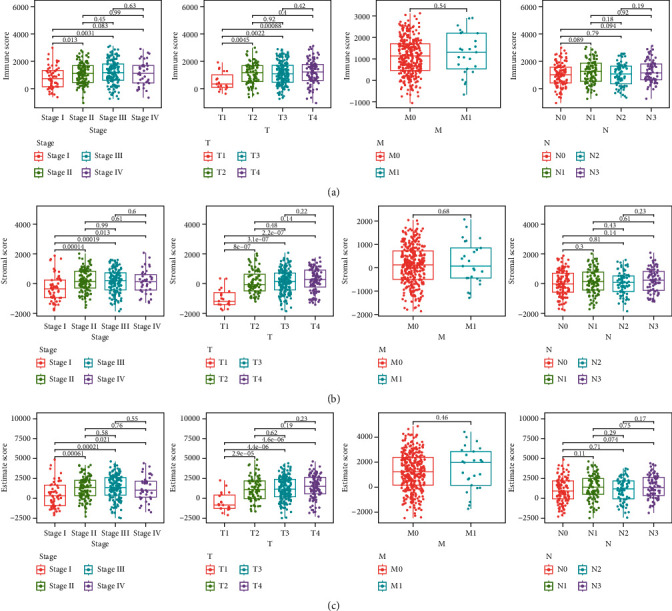
Association of StromalScore and ImmuneScore with clinical characteristics. Distribution of (a) ImmuneScore, (b) StromalScore, and (c) ESTIMATEScore in stage, T classification, M classification, and N classification.

In order to identify the association between the percentage of immunity and stroma constituents with the clinicopathology features, our team studied the relevant clinic data of GC sufferers from TCGA. As presented by Figure 2(a), the immunity value negatively exhibited an association with clinic phase and T categorization of TMN stages; StromalScore displayed a positive relationship with clinic phase and T categorization of TMN stages (Figure 2(b)), and ESTIMATEScore significantly declined accompanied with clinic phase and T categorization of TMN stages (Figure 2(c)).

### 3.3. DEGs between Smaller Immunity Value, Stroma Value and Greater Immunity Value, Stroma Value

**Figure 3:**
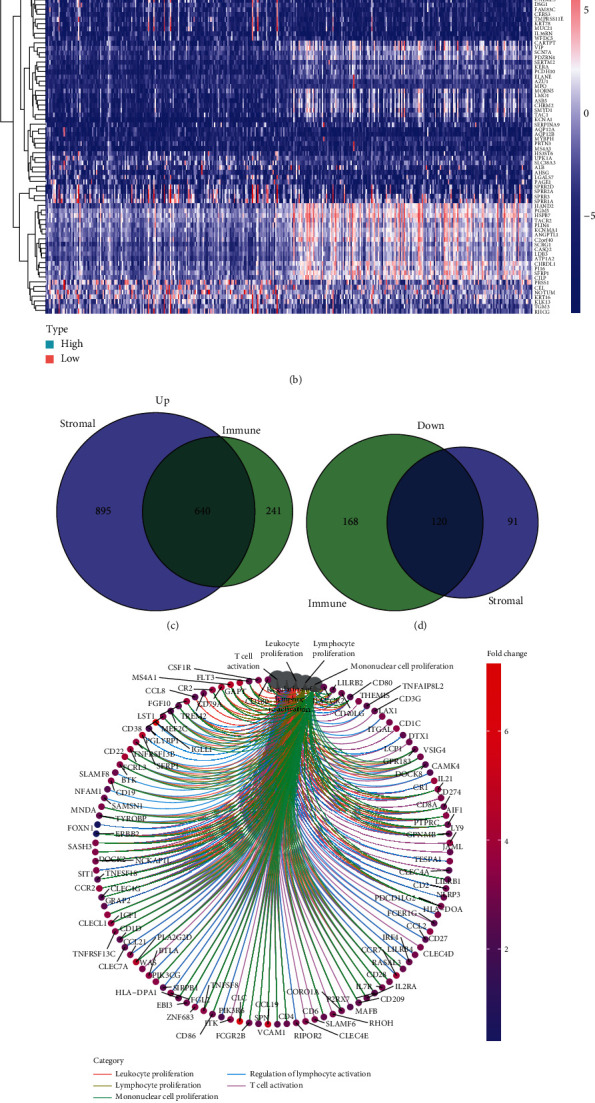
The expression pattern of DEGs and their enrichment analysis of GO and KEGG. (a) Heatmap for dysregulated genes in ImmuneScore. (b) Heatmap for dysregulated genes in StromalScore. (c, d) Venn plots of common dysregulated genes shared by ImmuneScore and StromalScore. (e, f) GO and KEGG enrichment analyses for 640 DEGs.

**Figure 4:**
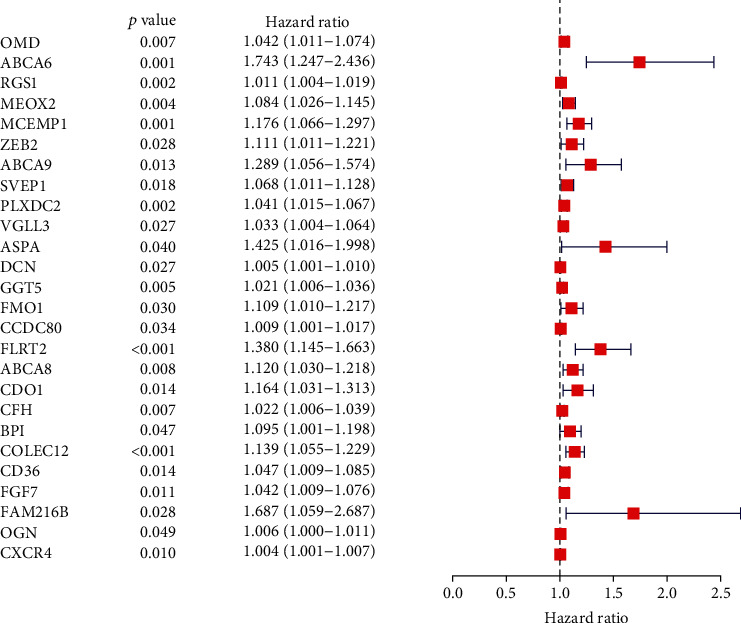
Univariate assays with 640 DEGs. The top distinct genes were listed.

To screen the dysregulated genes in GC, the levels of genes in the low and high score samples were studied. As shown in Figure 3(a), a total 1746 DEGs were acquired from stroma value (specimens with great value versus small value). Likewise, 1169 DEGs were acquired from the immunity value (Figure 3(b)). The results of intersection assays exhibited a total of 640 overexpressed genes sharing by great value both in immunity value and stroma value and 120 downregulated genes sharing by small value as well (Figures 3(c) and 3(d)). Results from GO enriching assay revealed that the DEGs nearly matched the immunity-associated GO terms, like white blood cell proliferative activities, lymphocyte proliferation, mononuclear cell proliferative activities, modulation of leukomonocyte stimulation, and T cell stimulation (Figure 3(e)). The KEGG enrichment assay also displayed the enrichment of virus protein mutual effect with cell factor and cell factor acceptor, NF-kappa B signal path, cytokine-cytokine acceptor interaction, chemokine signal path, and calcium signal path (Figure 3(f)). Moreover, univariable COX regressive assay for the survival of GC sufferers was completed to identify the important factors within 760 DEGs, and we identified 26 prognostic genes including OMD, ABCA6, RGS1, MEOX2, MCEMP1, ZEB2, ABCA9, SVEP1, PLXDC2, VGLL3, ASPA, DCN, GGT5, FMO1, CCDC80, FLRT2, ABCA8, CDO1, CFH, BPI, COLEC12, CD36, FGF7, FAM216B, OGN, and CXCR4 (Figure 4).

### 3.4. The Expressing and Clinic Importance of PLXDC2 in GC Patients

**Figure 5:**
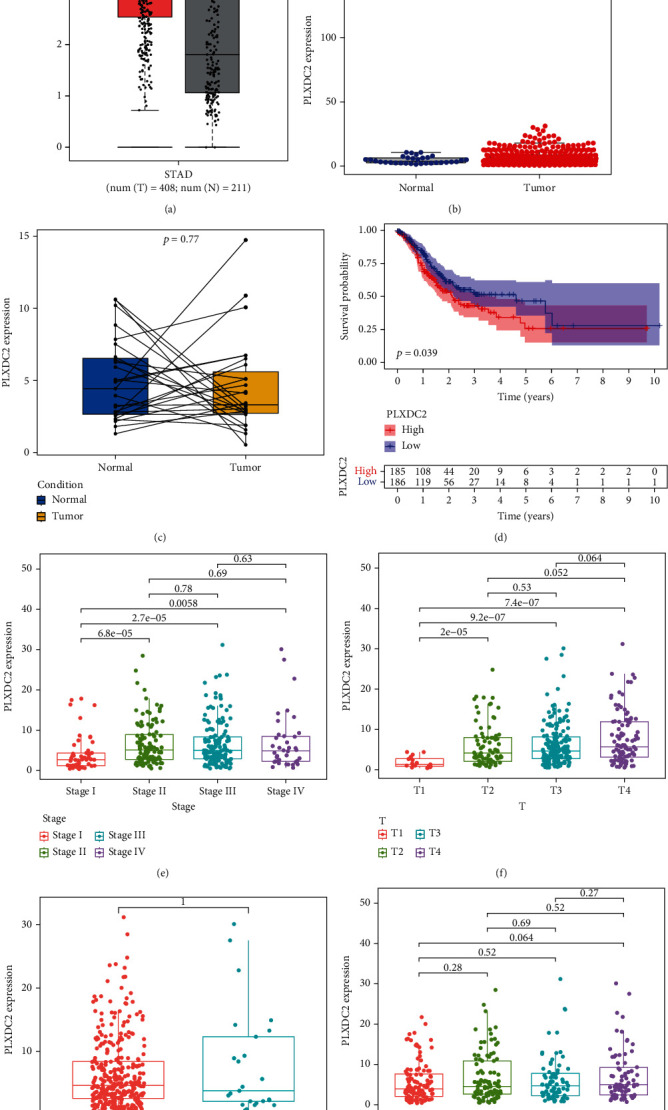
The expression of PLXDC2 in GC and its clinical significance. (a) The upregulation of PLXDC2 in GC specimens using TCGA and GTEx datasets. (b) No significance was observed just using TCGA datasets. (c) The levels of PLXDC2 in a paired differentiation analysis. (d) Survival assays of 371 GC patients based on the mean expression of PLXDC2. (e–h) The expression of PLXDC2 in stage (e), T classification (f), M classification (g), and N classification (h), ^∗^*p* < 0.05.

Among the 26 prognostic genes, we focused on PLXDC2. Compared with PLXDC2 expressions, the PLXDC2 expressions of healthy individuals were remarkably weaker in contrast to GC sufferers based on the TCGA and GTEx datasets (Figure 5(a)). However, there is no distinct difference between PLXDC2 expression and tumor specimens just based on the TGCA datasets (Figures 5(b) and 5(c)). Moreover, survival assays revealed that sufferers with remarkable PLXDC2 expressing predicted an inferior OS of GC patients (Figure 5(d), *p* = 0.039). Moreover, we observed that the expression of PLXDC2 in TME was positively related to the prognostic results of GC sufferers, particularly in phase and T categorization (Figures 5(e)–5(h)).

### 3.5. PLXDC2 Might Be an Underlying Marker of TME Regulation

**Figure 6:**
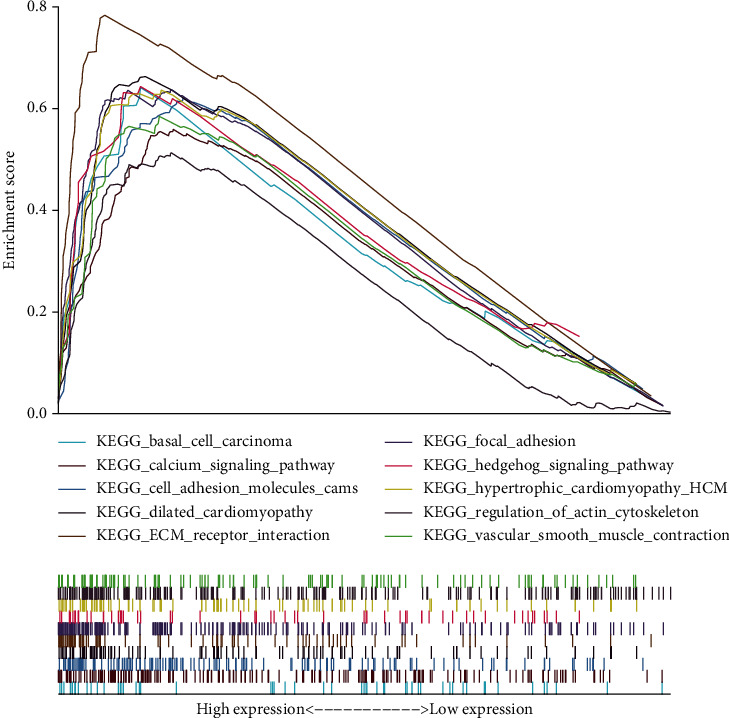
GSEA for cases with high PLXDC2 expressions and low expressions.

In contrast to the medium levels of PLXDC2, GESA of PLXDC2 in the high and low expressing groups was implemented. As presented by Figure 6, the genes in the PLXDC2 high expressing group were primarily gathered in immunity-associated events, like basal cell carcinoma, calcium signal path, cell adhesion molecules cams, dilated cardiomyopathy, acceptor mutual effect, macula adherens, Hedgehog signal path, hypertrophic cardiomyopathy, modulation of actin cellular skeleton, and vessel smooth muscle constriction. It revealed that PLXDC2 might be an underlying marker of TME conditions.

### 3.6. Association of PLXDC2 with the Percentage of TICs

**Figure 7:**
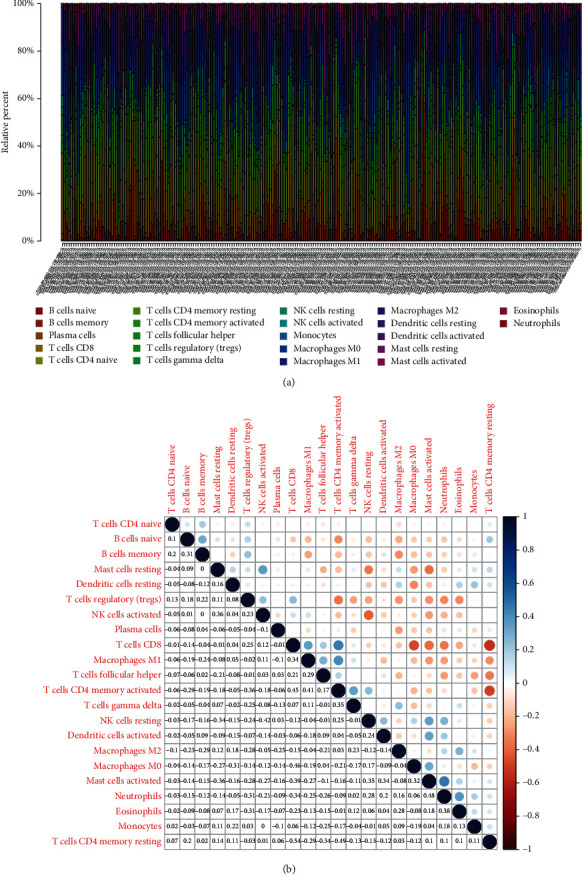
TIC profiles in cancer specimens and correlation assays. (a) Bar plot showing the proportion of 21 kinds of TICs in GC cases. (b) Heatmap of the correlations between 21 kinds of TICs and numeric.

**Figure 8:**
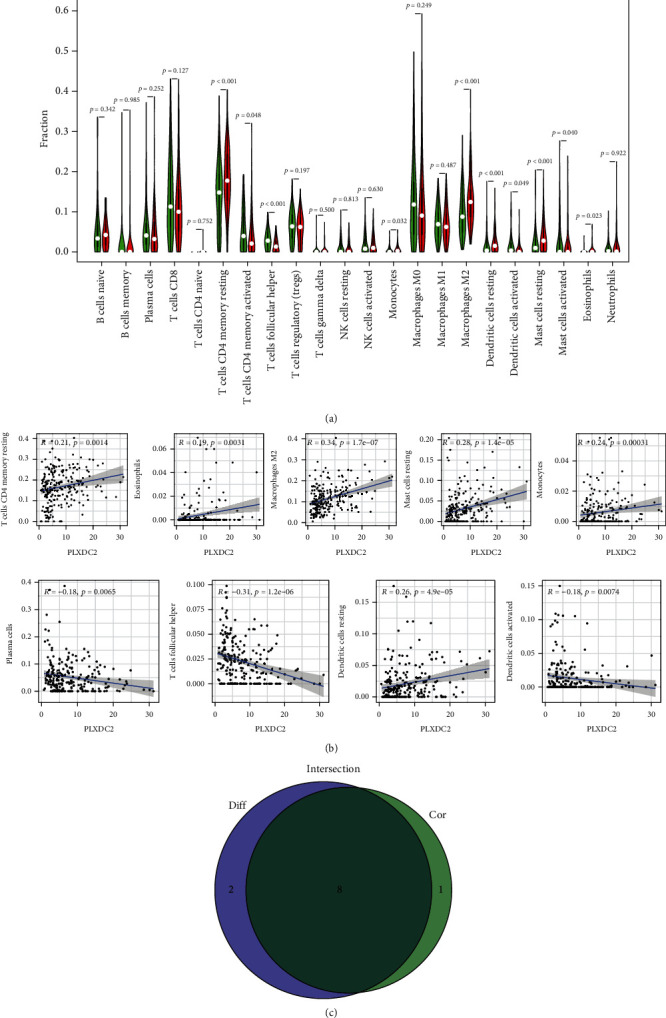
Associations of TICs proportion with PLXDC2 expressions. (a) Violin plot showed the ratio differentiation of 21 kinds of immune cells between GC samples with high PLXDC2 expressions and low PLXDC2 expressions. (b) Scatter plot showed the association of 8 kinds of TICs proportion with the PLXDC2 expressions. (c) Venn plot exhibited eight kinds of TICs related to PLXDC2 expressions.

For the purpose of confirming the association of PLXDC2 expressions with the immunity microenvironmental status, the percentage of tumor infiltrating immunity subsets was assayed via CIBERSORT arithmetic, and 21 types of immunocyte profiles in GC specimens were established (Figures 7(a) and 7(b)). The results showed that T cell CD4 memory resting, eosinophils, macrophage M2, mastocyte resting, mononuclear cells, and dendritic cell resting of highly expressed group of PLXDC2 was remarkably greater in contrast to the low expressing group of PLXDC2; the plasma cells, T cell follicle helper, and dendritic cells stimulated of highly-expressed group of PLXDC2 was remarkably lower in contrast to the low expressing group of PLXDC2 (Figure 8(a)). In addition, there were remarkable association between PLXDC2 expressing and the percentage of T cell CD4 memory resting, eosinophils, macrophage M2, mastocyte resting, monocytes, dendritic cell resting, the plasma cells, T cell follicle helper, and dendritic cells activated (Figure 8(b)).  Figure 8(c) showed intersection outcomes from the diversity and association assay, which unveiled that 8 kinds of TICs were remarkably related to the PLXDC2 expression.

### 3.7. The Oncogenic Roles of PLXDC2 in GC

**Figure 9:**
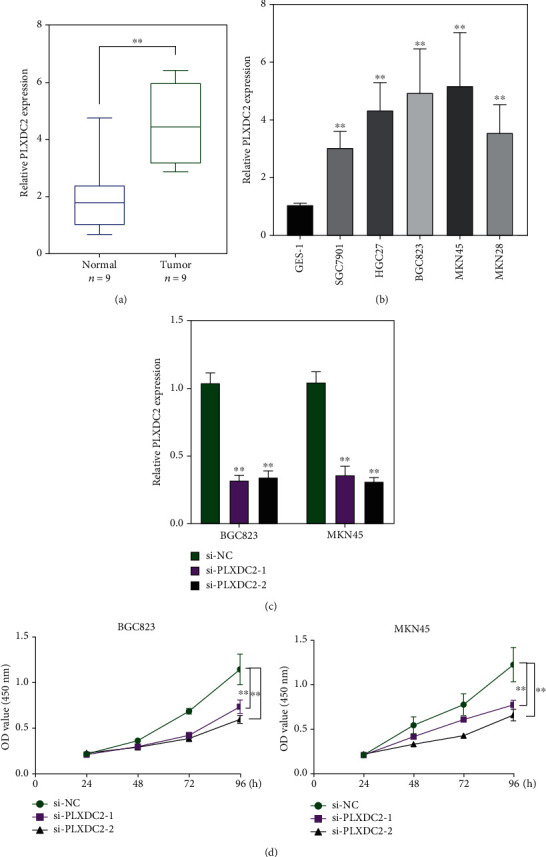
The increased expression of PLXDC2 and its oncogenic roles. (a) RT-PCR for the expression of PLXDC2 in 9 pairs of GC specimens and matched nontumor specimens. (b) The levels of PLXDC2 in five GC cells were determined by RT-PCR. (c) RT-PCR confirmed the transfection efficiency of si-PLXDC2-1 and si-PLXDC2-2 in BGC823 and MKN45 cells. (d) Cell proliferation ability was compared between the PLXDC2 siRNA stable transfection and negative control in BGC823 and MKN45 cells, ^∗∗^*p* < 0.01.

To demonstrate the levels of PLXDC2 in GC patients, we collected performed RT-PCR, finding that PLXDC2 expression was distinctly increased in GC specimens compared with matched nontumor specimens (Figure 9(a)). We also observed that PLXDC2 expressions were distinctly upregulated in five GC cell lines compared with GES-1 (Figure 9(b)). Moreover, to study the function of PLXDC2 in GC progression, we silenced PLXDC2 expression, which was demonstrated by RT-PCR (Figure 9(c)). Further, CCK-8 assays showed that knockdown of PLXDC2 suppressed the proliferation of BGC823 and MKN45 cells (Figure 9(d)).

## 4. Discussion

GC is one of the most commonly seen and aggressive mankind malignant cancers across the globe [22]. Although there has been advancement in surgical operations and auxiliary chemoradiation therapy, the 5-year OS ratio for GC sufferers improves remarkably with cancer development [23, 24]. TME was pivotal for the onset and development of carcinogenesis [25]. It is quite favorable to investigate the underlying treatment targets facilitating the reconstruction of TME and promoting the conversion of TME. Masses of researches highlighted the significance of immunity microenvironmental status in carcinogenesis [26, 27]. The outcomes herein from the transcription group assay percentage to GC information in TCGA revealed that the immunity constituents in TME facilitated the prognostic results of sufferers [28, 29]. Our evidences highlighted the significances of investigating the interaction between oncocytes and immunocytes, which offered novel enlightenment for designing more valid therapeutic strategies. As per the aforementioned speculation, our team found that a remarkable stroma value could be an underlying marker of an inferior prognostic result for GC sufferers.

For the purpose of understanding the precise variations of genetic profile in TME with regard to immunity and stroma constituents, the contrast assay between the high- and low-score specimens was completed. We identified a total of 640 upregulated genes sharing by a great score both in ImmunityScore and StromaScore and 120 downregulation genes sharing a small score as well. Then, we performed GO enriching assay and found that the DEGs nearly matched the immunity-associated GO terms, like white blood cell proliferative activities, lymphocyte proliferation, mononuclear cell proliferative activities, modulation of leukomonocyte stimulation, and T cell stimulation. Moreover, KEGG assays also displayed the enrichment of a cytokine receptor, NF-kappa B signal transmission, cell factor-cell factor acceptor mutual effect, and calcium signal transmission. Our finding suggested that the general roles of DEGs appeared to match immunity-associated events, which revealed that the participation of immunity factors marked a primary characteristic of TME in GC.

In recent years, more and more immune-related genes were reported to be related to clinic results and progression of GC sufferers [30, 31]. For instance, TGF*β*2, an essential regulator of immune cell functionality, was reported highly expressed in GC specimens and predicted a shorter overall survival of GC patients [32]. Lin et al. showed that CXCL8 expression was distinctly upregulated in GC and indicated poor clinical outcome of GC patients. Great proportion of CXCL8 is related to reduced CD8+ T cell infiltrative activities and Ki67+ CD8+ T cell percentage. M2 macrophage-secreted CHI3L1 was shown to promote the metastatic activities of GC cells in vitro and in vivo via interaction with IL-13R*α*2 [33]. These findings highlighted the potential of immune-related genes utilized as novel diagnosis and prognosis markers for GC patients. In this study, univariable COX regressive assay for the survival of GC sufferers was completed to identify the important factors within 760 DEGs, and we identified 26 prognostic genes, including OMD, ABCA6, RGS1, MEOX2, MCEMP1, ZEB2, ABCA9, SVEP1, PLXDC2, VGLL3, ASPA, DCN, GGT5, FMO1, CCDC80, FLRT2, ABCA8, CDO1, CFH, BPI, COLEC12, CD36, FGF7, FAM216B, OGN, and CXCR4. We analyzed the above genes and found many of them have been studied in many tumors, including GC [34–37]. In addition, their expression did not show distinct dysregulation between GC specimens and nontumor specimens based on the TCGA datasets. However, we found a specific gene PLXDC2. Based on the TGCA dataset, we found its expression in GC specimens was not different from normal gastric specimens. However, when we analyzed the samples from the TCGA datasets plus Genotype-Tissue Expression (GTEx) databases, the distinct upregulation of PLXDC2 was observed in GC specimens compared with nontumor specimens, which was also demonstrated in our cohort. Functional assays indicated that knockdown of PLXDC2 suppressed the proliferation of GC cells. Besides, the effects of PLXDC2 in GC were rarely reported. Clinical assays confirmed that high PLXDC2 expression was related to shorter survivals, late period clinically, and T stage. The GSEA outcomes revealed that immunity-associated signal path, like basal cell carcinoma, calcium signal path, cell adhesion molecules cams, dilated cardiomyopathy, acceptor mutual effect, macula adherens, Hedgehog signal path, hypertrophic cardiomyopathy, modulation of actin cellular skeleton, and vessel smooth muscle constriction, were remarkably gathered in the PLXDC2 highly expressed group. Those outcomes revealed that PLXDC2 might be involved in the status transformation of TME from immunity dominance to metabolism dominance.

Recently, an immune checkpoint inhibitor (ICI) has become one of the fastest progressing immune treatment methods of GC [38, 39]. ICI is able to blockade cancer-triggered immune suppression, hence reinforcing the anticancer immunoresponse [40]. Immunocheckpoints are suppressive cancer immunity acceptors, which are located on the surface of stimulated T cells [41]. After the immunocheckpoint is combined with the cancer superficial antigen, it is able to suppress cancer immunoresponse and facilitate cancer immunoescape [42, 43]. In this study, we found that T cell CD4 memory resting, eosinophils, macrophage M2, mastocyte resting, mononuclear cells, and dendritic cell resting of highly expressed group of PLXDC2 were remarkably greater in contrast to the low expressing group of PLXDC2; the plasma cells, T cell follicle helper, and dendritic cells stimulated of highly expressed group of PLXDC2 was remarkably lower in contrast to the low expressing group of PLXDC2. These results revealed that the influence of PLXDC2 expressing on the immunoactivity of TME.

## 5. Conclusion

Our team identified the TME-associated genes in GC via ESTIMATE arithmetic in the TCGA database. PLXDC2 expression was distinctly increased in GC and may be a possible biomarker for GC patients. A remarkable association existed between PLXDC2 expression and the percentage of T cell CD4 memory resting, eosinophils, macrophage M2, mastocyte resting, monocytes, dendritic cell resting, plasma cells, T cell follicle helper, and dendritic cells activated. The signature herein may indicate that PLXDC2 is an underlying biomarker for the prognostic results of GC patients and it is related to immune infiltration.

## Data Availability

The datasets used and analyzed during the current study are available from the corresponding author on reasonable request.

## Abbreviations

ImmPort: Immunology Database and Analysis Portal
FDR: False discovery rate
FC: Fold change
OS: Overall survival
IL-13R*α*2: Interleukin-13 receptor *α*2 chain
ICI: Immune checkpoint inhibitor.
